# Phytaspase Does Not Require Proteolytic Activity for Its Stress-Induced Internalization

**DOI:** 10.3390/ijms25126729

**Published:** 2024-06-19

**Authors:** Tatevik A. Torosian, Anastasia I. Barsukova, Nina V. Chichkova, Andrey B. Vartapetian

**Affiliations:** 1 Faculty of Bioengineering and Bioinformatics, Lomonosov Moscow State University, Moscow 199991, Russia; tatoshik312@gmail.com (T.A.T.); nastiabarsukova@gmail.com (A.I.B.); 2 Belozersky Institute of Physico-Chemical Biology, Lomonosov Moscow State University, Moscow 199991, Russia; chic@belozersky.msu.ru

**Keywords:** endocytosis, oxidative stress, phytaspase, plant cell, prodomain, protein trafficking, proteolytic activity, subtilisin-like protease

## Abstract

Phytaspases differ from other members of the plant subtilisin-like protease family by having rare aspartate cleavage specificity and unusual localization dynamics. Phytaspases are secreted from healthy plant cells but are re-internalized upon perception of death-inducing stresses. Although proteolytic activity is required for the secretion of plant subtilases, its requirement for the retrograde transportation of phytaspases is currently unknown. To address this issue, we employed an approach to complement in trans the externalization of a prodomain-less form of *Nicotiana tabacum* phytaspase (*Nt*Phyt) with the free prodomain in *Nicotiana benthamiana* leaf cells. Using this approach, the generation of the proteolytically active *Nt*Phyt and its transport to the extracellular space at a level comparable to that of the native *Nt*Phyt (synthesized as a canonical prodomain-containing precursor protein) were achieved. The application of this methodology to *Nt*Phyt with a mutated catalytic Ser537 residue resulted in the secretion of the inactive, although processed (prodomain-free), protein as well. Notably, the externalized *Nt*Phyt Ser537Ala mutant was still capable of retrograde transportation into plant cells upon the induction of oxidative stress. Our data thus indicate that the proteolytic activity of *Nt*Phyt is dispensable for stress-induced retrograde transport of the enzyme.

## 1. Introduction

**Figure 1:**
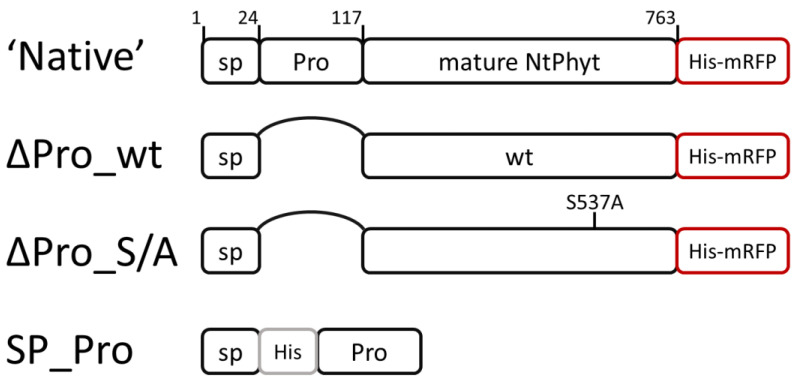
Schematic representation of *Nt*Phyt derivatives constructed in this study. ‘Native’, intact *Nt*Phyt precursor C-terminally tagged with His_6_-mRFP served as a positive control of *Nt*Phyt localization and proteolytic activity. Prodomain region (Pro) was precisely deleted from this protein to produce ∆Pro_wt construct and from the catalytically inactive Ser537Ala mutant precursor protein to produce ∆Pro_S/A. SP – signal peptide. SP_Pro construct used to complement the prodomain deficiency contains a His_6_ insertion 2 amino acid residues downstream from the signal peptidase cleavage site (as determined in [4]) to permit its detection using Western blot analysis. Boundaries of the mature endogenous *Nt*Phyt and position of the Ser537Ala mutation are indicated.

The localization of proteins to proper cellular compartments is crucial for their action and for cell viability. This applies equally to plant serine proteases, the largest family of which comprises subtilisin-like proteases (subtilases). Subtilases contribute to various aspects of growth and development in plants [1,2], and the vast majority of these enzymes is extracellular. This is due to the presence of an N-terminal signal peptide in their precursor proteins (see Figure 1 for schematic representation) that targets subtilase preproenzymes to the endoplasmic reticulum. Upon the subsequent journey to the cell exterior, subtilase proenzymes become constitutively activated through the autocatalytic detachment of the prodomain (Figure 1), and mature enzymes are eventually secreted into the apoplast [1]. Although the apoplast has long been considered to be the end point of the subtilase transport pathway, for a group of plant subtilisin-like subtilases termed phytaspases, a continuation of the route was recently discovered [3]. Phytaspases, being structurally typical subtilisin-like proteases, derive their name from their strict substrate cleavage specificity after an aspartate residue preceded by a characteristic amino acid motif [4,5,6], strongly resembling the substrate specificity of animal apoptotic proteases (caspases) [7,8,9]. Also, like caspases, phytaspases are involved in the execution of cell death induced by biotic and abiotic stresses [4,10,11,12]. Unexpectedly, death-inducing stimuli trigger the specific retrograde transport of mature and proteolytically active phytaspases from the apoplast to the cell interior [3,4], thus providing these enzymes with access to intracellular protein targets. To re-enter the stressed cells, phytaspases utilize clathrin-mediated endocytosis [3,13]; however, the molecular interactions allowing these soluble extracellular enzymes to become endocytosed still remain unknown.

The intrinsic proteolytic activity of phytaspase (and of plant subtilisin-like proproteases in general) is clearly essential for the anterograde transport (secretion) of the native enzyme to the apoplast, as the impairment of autocatalytic detachment of the prodomain causes the proenzyme to become trapped within the cell [4,14]. In this study, we aimed to address a complementary question: whether or not the specific proteolytic activity of *Nicotiana tabacum* phytaspase (*Nt*Phyt) is required for stress-induced retrograde transport of the enzyme. By using an inactive, although processed, *Nt*Phyt mutant in combination with an in trans prodomain complementation approach [15,16], we were able to demonstrate that *Nt*Phyt lacking proteolytic activity is still competent for stress-induced internalization in *Nicotiana benthamiana* plants.

## 2. Results

### 2.1. Setup of the Phytaspase Transport Assay

To determine whether the proteolytic activity of *Nt*Phyt is required for stress-induced retrograde transport of the enzyme, an obvious approach would be to compare the behavior of the inactive enzyme (e.g., with mutated catalytic Ser^537^ residue [4]) with that of the wild-type counterpart. This seemingly ‘simple’ approach is, however, complicated by the fact that the catalytically inactive subtilase precursor protein would not be able to cleave off its prodomain and thus would not be transported to the apoplast [4,14,17], let alone allow retrograde transport to be investigated. On the other hand, a straightforward artificial deletion of the prodomain from the inactive phytaspase precursor is also not an option. In subtilases, the prodomain is anticipated to perform dual roles: it acts as an intramolecular chaperone for proenzyme folding and as an inhibitor of the mature enzyme [16,17,18,19]. Hence, one would expect that a phytaspase precursor protein lacking the prodomain is unlikely to pass the quality control within the endoplasmic reticulum, which in turn will preclude its secretion. However, the available data for bacterial subtilisin and for some plant subtilases indicated that production of the respective prodomain in trans (that is, in the same cell with the prodomain-deficient subtilase precursor) could complement the defect in subtilase folding and secretion, at least partially [15,16].

Taking into account the above considerations, several derivatives of the *Nt*Phyt preproenzyme were constructed, alongside a non-modified precursor protein containing a His_6_-mRFP (monomeric red fluorescent protein) tag at its C-terminus (Figure 1, ‘native’). In ∆Pro_wt and ∆Pro_S/A proteins comprising the signal peptide and the catalytic domain of the wild-type enzyme and of the S537A inactive mutant, respectively, the prodomain regions were precisely deleted (Figure 1, ∆Pro_wt and ∆Pro_S/A), guided by the previously established processing sites within the *Nt*Phyt precursor [4]. To enable detection, these phytaspase derivatives were also C-terminally tagged with His_6_-mRFP, a modification that was shown previously not to interfere with proteolytic activity and trafficking of *Nt*Phyt and to be relatively stable within the aggressive apoplast environment [3,4]. An SP-Pro construct encoding the *Nt*Phyt signal peptide linked to the prodomain via a His_6_ spacer was also made (Figure 1).

### 2.2. Elimination of the Prodomain from the NtPhyt Precursor Compromises Proteolytic Activity and Secretion of NtPhyt

**Figure 2:**
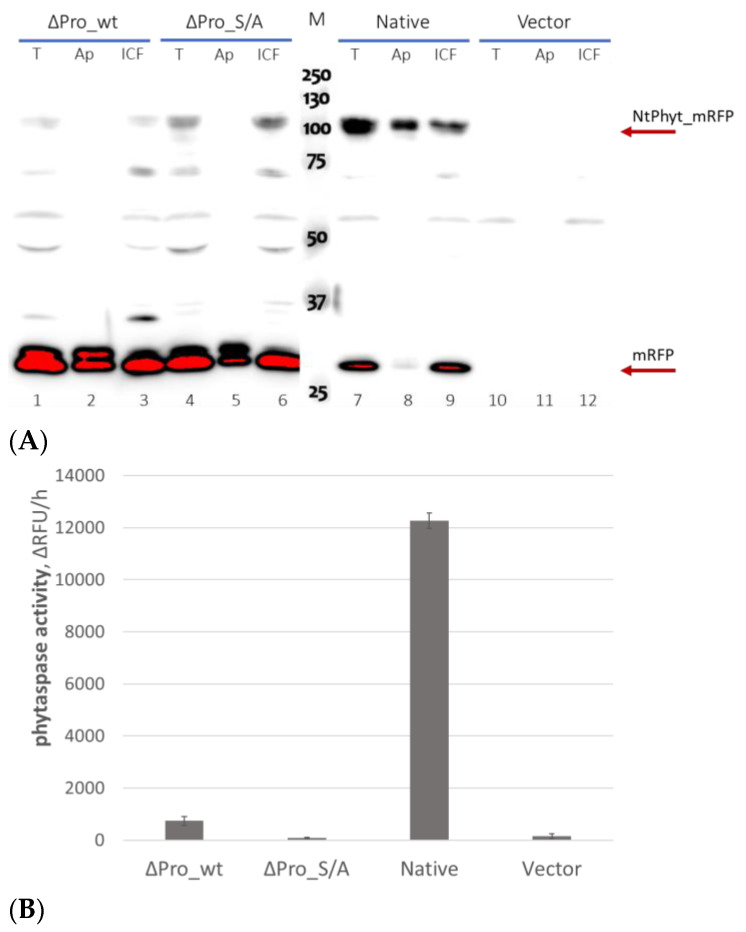
The prodomain-less version of the *Nt*Phyt precursor protein displays impaired proteolytic activity and is not exported into the apoplast. (**A**) Relative levels of ‘native’, ∆Pro_wt, and ∆Pro_S/A proteins tagged with mRFP were evaluated in the intracellular (ICF), apoplastic (Ap), and total (T) protein fractions from *N. benthamiana* leaves transiently producing the respective protein. Protein fractionation was performed at 3 days post-infiltration (dpi), using equal weight amounts of leaves. Proteins were separated with 10% SDS–polyacrylamide gel electrophoresis (PAGE). Blot was developed using anti-mRFP antibodies and peroxidase-conjugated secondary antibodies with enhanced chemiluminescence detection. Vector, protein fractions from leaves infiltrated with agrobacteria carrying the empty vector. M, MW protein markers. Arrows indicate the position of the *Nt*Phyt-mRFP recombinant protein (MW ~ 110 kDa) and of free mRFP. Note that the intensity of an intracellular protein band (~55 kDa) non-specifically recognized by anti-mRFP antibodies may serve as a loading control. (**B**) Measurement of phytaspase proteolytic activity in total leaf extracts (equivalent to those analyzed in lanes 1, 4, 7, and 10 in (**A**)), using a fluorogenic peptide substrate, Ac-VEID-AFC (20 μM). Relative rates of hydrolysis were determined as an increase in relative fluorescence units per hour (∆RFU/h). Data represent the mean of three experiments ± standard deviation (SD).

The integrity and localization of the prodomain-less versions of *Nt*Phyt and of the ‘native’ *Nt*Phyt precursor were compared. To this end, the recombinant proteins were produced in *N. benthamiana* leaves by means of agroinfiltration. Total, apoplastic, and intracellular protein fractions obtained from these leaves were analyzed by Western blotting with anti-mRFP antibodies. The ‘native’ *Nt*Phyt demonstrated remarkable stability, as inferred from the presence of the full-length (~110 kDa) protein band, and a low level of degradation, as manifested by the intensity of a free mRFP band (~30 kDa) (Figure 2A, lanes 7–9). As expected, the enzyme was clearly detectable in the apoplast. In contrast to this behavior, the prodomain-less versions of *Nt*Phyt were found to be far less stable, and neither the prodomain-less wild-type protein (∆Pro_wt) nor the ∆Pro_S/A mutant could be detected in the apoplastic fraction (Figure 2A, lanes 1–3 and 4–6, respectively).

To verify the results of the blotting experiment, *Nt*Phyt proteolytic activity in the above-described total leaf extracts was determined using an *Nt*Phyt-specific fluorogenic peptide substrate, Ac-VEID-AFC [4]. Consistent with the previous data, a high level of *Nt*Phyt activity was observed in the ‘native’ *Nt*Phyt-producing leaves, whereas the activity of the ∆Pro_wt and ∆Pro_S/A samples was low (Figure 2B). Interestingly, although some increase in the *NtPhyt* proteolytic activity was observed for the ∆Pro_wt-producing (but not for the ∆Pro_S/A-producing) leaves relative to the vector control, this activity was still markedly lower than that of the ‘native’ sample (Figure 2B).

Thus, the elimination of the prodomain from the *Nt*Phyt precursor compromised the proteolytic activity of the wild-type enzyme and prevented the secretion of both the ‘wild-type’ and the mutant S/A versions of prodomain-less phytaspase into the apoplast.

### 2.3. Rescuing the Prodomain-Less NtPhyt

**Figure 3:**
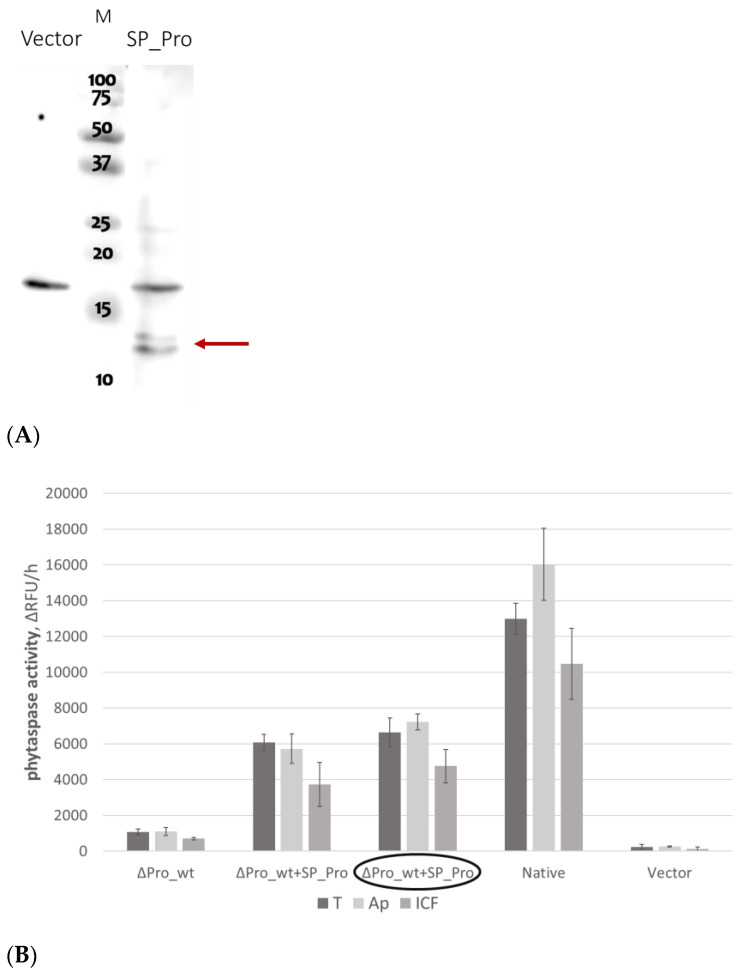

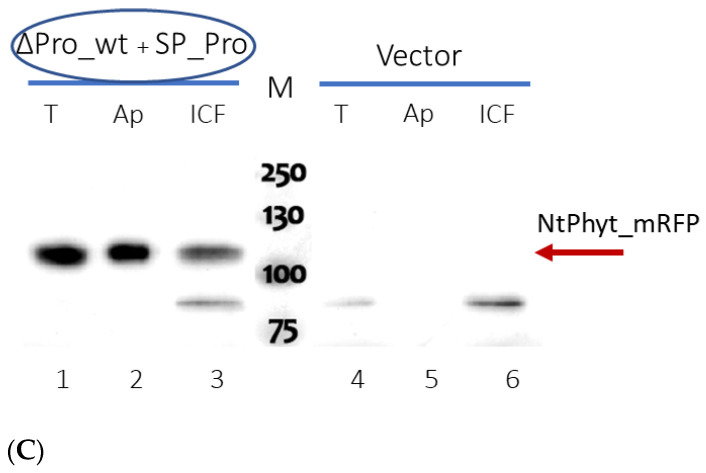
Co-production of ∆Pro_wt with SP_Pro allows for the formation of proteolytically active phytaspase and its secretion. (**A**) Transient production of SP_Pro in *N. benthamiana* leaves. Total protein extracts from SP_Pro-producing and control (vector) leaves were fractionated by 15% SDS-PAGE and analyzed by Western blotting with HisProbe detection. The position of the recombinant protein is indicated (arrow, ~12 kDa). SP_Pro forms a double band, likely representing the prodomain with the non-cleaved and with cleaved off signal peptide (SP). M, MW protein markers. (**B**) Determination of phytaspase proteolytic activity in the total (T), apoplastic (Ap), and intracellular (ICF) protein fractions obtained from leaves infiltrated with agrobacteria bearing the ∆Pro_wt-encoding plasmid only (∆Pro_wt), with a mixture of agrobacteria bearing the ∆Pro_wt and the SP_Pro-encoding plasmid (∆Pro_wt + SP_Pro), with double-transformed agrobacteria (∆Pro_wt + SP_Pro, circled), with agrobacteria bearing the ‘native’ construct (‘Native’), and of control extracts (Vector). Ac-VEID-AFC peptide (20 μM) was used as a fluorogenic phytaspase substrate. Enzymatic activities (expressed as ∆RFU/h) were normalized by the weight of leaf tissues taken for analysis. Data represent the mean of three experiments ± SD. (**C**) Western blot analysis, with anti-mRFP antibodies of protein fractions (the same as in (**B**)) demonstrating complementation of the ∆Pro_wt secretion in the presence of SP_Pro. Protein extracts were separated with 12% SDS–gel electrophoresis. M, MW protein markers.

The co-production of a prodomain with a prodomain-less subtilase was previously reported to partially improve the intracellular stability and secretion of the latter [16]. To test the feasibility of this approach with respect to *Nt*Phyt, we first tested the co-production of SP_Pro protein (Figure 3A) with the prodomain-less non-mutated *Nt*Phyt precursor, ∆Pro_wt, by infiltrating leaves with a mixture of agrobacteria carrying plasmids encoding each of the proteins. Alternatively, we placed the respective genes (encoding ∆Pro_wt and SP_Pro) in compatible expression vectors with distinct antibiotic resistance markers (see Section 4.1 for details) and obtained double-transformed agrobacterium cells carrying both protein-encoding plasmids simultaneously. An increase in *Nt*Phyt proteolytic activity in the apoplastic and intracellular fractions of the thus infiltrated leaves was taken as a criterion for the success of the approach.

In both cases, a marked increase in the *Nt*Phyt proteolytic activity in extracts of agroinfiltrated *N. benthamiana* leaves was achieved (Figure 3B). Most importantly, in the presence of the prodomain, the majority of *Nt*Phyt activity was detected in the apoplastic fraction. Consistent with the *Nt*Phyt activity determination, the Western blot analysis of these fractions demonstrated significant accumulation of the secreted ∆Pro_wt enzyme in the apoplast (Figure 3C, lane 2).

We thus conclude that we succeeded in making the prodomain-less version of wild-type *Nt*Phyt secretable.

### 2.4. Catalytically Inactive NtPhyt Mutant Is Competent for Stress-Induced Internalization

**Figure 4:**
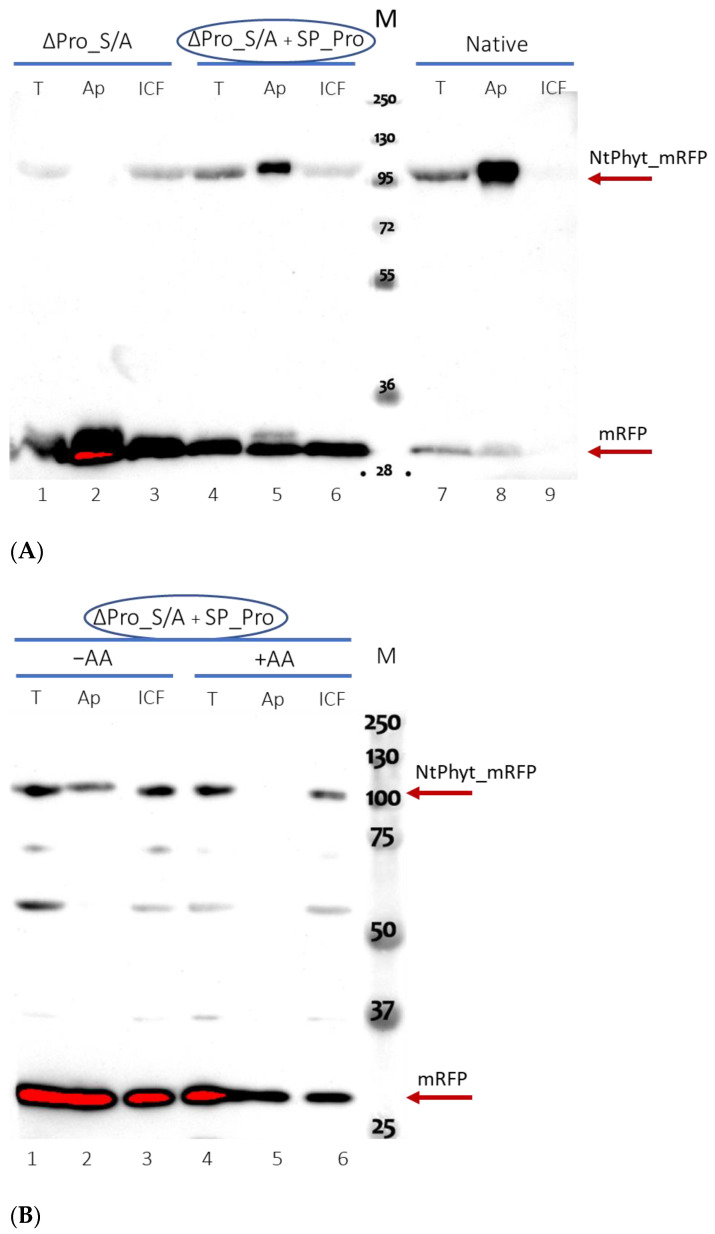
Localization dynamics of the catalytically inactive *Nt*Phyt. (**A**) Distribution between the apoplastic (Ap) and intracellular (ICF) protein fractions of ∆Pro_S/A protein produced in *N. benthamiana* leaves either alone or in combination with SP_Pro, using double-transformed agrobacteria. T, total protein. The analogous protein fractions from leaves producing the ‘native’ construct serve as controls. Protein samples were separated with 10% SDS_PAGE and visualized by Western blot analysis with anti-mRFP antibodies. M, MW protein markers. (**B**) Oxidative stress-induced internalization of the proteolytically inactive *Nt*Phyt mutant. *N. benthamiana* leaves were infiltrated with agrobacteria double transformed with the ∆Pro_S/A and SP_Pro-encoding plasmids. At 3 dpi, a portion of leaf tissues was subjected to oxidative stress by treatment with 40 μM antimycin A for 5 h (+AA), while the other portion was mock-treated (−AA) (see Section 4.3 for details). The apoplastic (Ap), intracellular (ICF), and total (T) protein fractions were obtained from equal-weight amounts of lief pieces of each type and separated by 10% SDS-PAGE. Western blot analysis was performed using anti-mRFP antibodies. M, MW protein markers. Arrows indicate the positions of *Nt*Phyt-mRFP and free mRFP proteins. Data were reproducible over three independent experiments.

We then applied the same strategy to the force secretion of the inactive ∆Pro_S/A *Nt*Phyt derivative into the apoplast of infiltrated *N. benthamiana* leaves. As the activity assay could not be applied to the S/A mutant [4] (see also Supplementary Figure S1), a Western blot analysis of the intracellular and apoplastic protein fractions was used to evaluate secretion. Figure 4A shows that the approach turned out to be successful, resulting in significant secretion of the ∆Pro_S/A protein into the apoplast, whereas the same protein synthesized in the absence of the SP-Pro co-production was excluded from the apoplast. Of note, the inactive ∆Pro_S/A *Nt*Phyt derivative demonstrated the level of accumulation and stability in the apoplast similar to those of its wild-type counterpart (compare Figure 3C and Figure 4A).

Finally, to learn whether or not the extracellularly localized *Nt*Phyt mutant devoid of proteolytic activity is competent for stress-induced retrograde transportation, leaf discs containing the externalized ∆Pro_S/A protein were subjected to oxidative stress via treatment with 40 μM antimycin A [13]. A comparison of intra- and extracellular protein fractions by Western blot revealed the translocation of the inactive phytaspase from the apoplast toward the cell interior, as compared to the mock-treated control (Figure 4B).

We interpret these findings to indicate that the retrograde transport of *Nt*Phyt in response to stress does not require the proteolytic activity of the phytaspase.

## 3. Discussion

Within the trafficking route of phytaspases, retrograde transport to deliver the active enzyme from the apoplast into the cell in response to cell death-inducing stress is the most unusual and, mechanistically, the least understood step. As phytaspases exist as soluble enzymes in the apoplastic fluid [4,12,13], their uptake into the cell by means of clathrin-mediated endocytosis appears to require a phytaspase-recognizing receptor at the plant cell surface to serve as an interface [3]. However, neither the receptor nor specific features of phytaspase essential for internalization/interaction with such a receptor are currently known.

Here, we demonstrated that the proteolytic activity of *Nt*Phyt is dispensable for its stress-induced uptake. In order to direct catalytically inactive, although processed, *Nt*Phyt to an extracellular location, an in trans complementation by the separately synthesized *Nt*Phyt prodomain was employed.

Given that *Nt*Phyt does not require its proteolytic activity for retrograde transport, other ways of phytaspase interaction with its putative receptor should be considered. From studies in animal cells, two types of protease–receptor interaction may provide instructive illustrations. The first one is exemplified by cell surface localized protease-activated receptors (PARs). PARs are G protein-coupled receptors that are activated by proteolysis [20,21,22]. Upon the binding of a cognate extracellular protease, PAR becomes proteolytically processed, generating a new N-terminal domain that binds to the receptor intramolecularly to induce intracellular signaling [23,24]. A wide range of PAR–protease pairs are currently known [20,25]. However, the protease–PAR interaction involves the activity of a protease and, besides, it was not documented to trigger the uptake of extracellular proteolytic enzyme.

Another example seems to be closer to the case of phytaspase. Subtilisin-like proteases (subtilases) in animals (also known as proprotein convertases) possess high selectivity of hydrolysis [26], although their cleavage specificity is different from that of phytaspases [27,28,29]. For one of these subtilases, PCSK9, which is an extracellular enzyme, binding to the complex formed by the low-density lipoprotein receptor (LDL-R) and its low-density lipoprotein cholesterol ligand was documented [30,31]. The formation of this stabilized ternary complex inhibits the recycling of the LDL-R back to the cell surface upon endocytosis; instead, it targets the complex toward the lysosomal compartment for degradation [30,31,32,33,34,35]. Notably, the engagement of PCSK9 in the formation of the complex with the LDL-R, as well as in the subsequent internalization and degradation of the complex, does not require the proteolytic activity of PCSK9 [36,37]. The lack of a requirement for proteolytic activity for the internalization of PCSK9 thus appears to be similar to that observed for phytaspase. However, a principal difference in the fates of the internalized animal and plant subtilases does exist. While PCSK9 complexed to LDL-R is targeted for degradation, no evident loss of proteolytic activity upon translocation was observed for phytaspase [3,13].

It would thus be important in the future to identify the hypothetical phytaspase receptor, as well as the mechanism that enables the endocytosed phytaspase to escape the degradative pathway.

## 4. Materials and Methods

### 4.1. Plant Growth Conditions

*N. benthamiana* plants were grown at 25 °C in soil with organic–mineral fertilizer (Gera Profi, Moscow Region, Russia) in a controlled environment, under a 16/8 h day/night cycle. For transient protein production, *Agrobacterium tumefaciens* C58C1 cells were transformed with the respective plasmid. Alternatively, cells double transformed with pCambia1300 and pLH7000 derivatives (see below), providing kanamycin resistance and streptomycin plus spectinomycin resistance, respectively, were obtained. To enhance the protein production, transformed cells were mixed with an equal number of agrobacteria bearing the p19 suppressor of silencing prior to infiltration. Agrobacteria carrying the empty vector were used as a control. Agrobacteria were infiltrated into leaves of 6-week-old plants using a blunt syringe. Typically, leaves were harvested 3 days post-infiltration (dpi).

### 4.2. Plasmid Construction

To create the ‘native’ *Nt*Phyt_His_6__mRFP fusion, the mRFP gene [13] was PCR amplified using Bam_His_mRFP_dir and mRFP_Sac(z)_rev primers (Supplementary Table S1) and inserted downstream of and in frame with the *Nt*Phyt-encoding sequence between the *Bam*HI and *Sac*I sites of the pLEX_NtPhyt_His plasmid [38] to obtain the pLEX_NtPhyt_His_mRFP plasmid. To generate the SP-protease domain junction, two DNA fragments were obtained by PCR on the pLEX_NtPhyt_His plasmid using pLH_seq_dir plus deltaPro_rev and deltaPro_dir plus sub_rev3-1 primer pairs. Subsequent amplification on the mix of these DNA fragments using external pLH_seq_dir and sub_rev3-1 primers [39] generated a 650 bp DNA fragment, which was treated with *Xho*I and *Pst*I and inserted between these sites of the pSL1180 vector to generate the pSL_SP_deltaPro_690 plasmid. Finally, the *Xho*I-*Pst*I DNA fragment from this plasmid (circa 550 bp) and the *Pst*I-*Bam*HI fragment (circa 1600 bp) from the pLEX_NtPhyt-His_mRFP plasmid were inserted, by triple ligation, between the *Xho*I and *Bam*HI sites of the pLEX_NtPhyt_His_mRFP plasmid to generate the pLEX_deltaPro_NtPhyt_His_mRFP plasmid.

To introduce the Ser537Ala mutation into thus obtained construct, the *Pst*I-*Bam*HI DNA fragment (circa 1600 bp) from the *Nt*Phyt cDNA Ser537Ala mutant [4] was first ligated between the *Pst*I and *Bam*HI sites of the pSL_SP_deltaPro_690 plasmid and then transferred, as the *Xho*I-*Bam*HI DNA fragment (circa 2100 bp), into the pLEX_NtPhyt-His_mRFP to substitute the wild-type *Nt*Phyt cDNA with the mutant one.

To create the SP_Pro fusion protein, three PCR amplification steps were performed. First, PCR on the pLEX_NtPhyt_His plasmid using SP_His_Pro_dir and Prodomain_Sac_rev primers generated a 320 bp cDNA fragment, which was subsequently elongated by PCR, using SP_middle_dir plus Prodomain_Sac_rev and SP_Nco_dir_ver2 plus Prodomain_Sac_rev primer pairs, respectively. The resultant 380 bp DNA fragment was treated with *Nco*I and *Sac*I, ligated between the corresponding sites of the pSL1180 vector and then further transferred into the pLEX7000 and pCambia1300EX binary vectors [13] to generate the pLEX_SP_His_Pro and pCambia_SP_His_Pro plasmids, respectively.

The identities of all constructs were confirmed using DNA sequence analysis. In planta expression of all constructed genes was driven by the dual cauliflower mosaic virus (CaMV) 35S promoter.

### 4.3. Transient Expression in N. benthamiana and Protein Fractionation

Leaf discs (approx. 15 mg each) from *N. benthamiana* plants transiently producing the recombinant proteins were prepared 3 dpi. Where indicated, treatment of leaf discs with antimycin A was performed by vacuum infiltration, with water containing 40 μM antimycin A (Sigma, St. Louis, MO, USA; from stock solution in ethanol) and incubation for 5 h in the dark. Control discs were infiltrated with distilled water supplemented with an equivalent amount of ethanol.

Leaf discs were subjected to protein fractionation for the subsequent determination of protein localization and phytaspase activity. To obtain the apoplastic and intracellular protein fractions, an approach shown previously to reliably separate intra- and extracellular proteins [13] was applied. Briefly, leaf pieces were vacuum infiltrated with MES100 buffer (20 mM MES, pH 5.5, and 100 mM NaCl) at the reduced pressure of 30 hPa (mbar) for 1 min, rolled into a Parafilm sheet, and centrifuged in a 1.5 mL centrifuge tube at 4 °C for 10 min at 2000× *g*. The apoplastic wash was collected from the bottom of the tube. The residual leaf material was frozen in liquid nitrogen and disrupted in Minilys homogenized (Bertin Instruments, Montigny-le-Bretonneux, France) using 1.4 mm ceramic beads by two 15 s bursts. An additional 20 s burst was performed after suspending the samples in B1 buffer (20 mM MES, pH 5.7, 50 mM NaCl, 2 mM DTT, 0.1% Tween 20, and 5% glycerol) supplemented with 500 mM NaCl, aprotinin (1.8 µg/mL), leupeptin (5.2 µg/mL), chymostatin (6 µg/mL), E64 (6 µg/mL), and 4-(2-aminoethyl)benzenesulfonyl fluoride (25 µg/mL) protease inhibitors. Debris was eliminated by 10 min centrifugation at 10,000× *g* at 4 °C, and the supernatants (as well as the apoplastic washes) were taken for phytaspase activity determination and for PAGE analysis of protein content. Leaves without prior separation of the apoplastic liquid were processed in an analogous fashion to obtain ‘total protein’ samples.

### 4.4. Phytaspase Activity Determination

The proteolytic activity of phytaspase in apoplastic washes, intracellular protein fractions, and total *N. benthamiana* leaf extracts was determined using *Nt*Phyt-specific Ac-VEID-AFC [AFC, 7-amino-4-(trifluoromethyl) coumarin] (California Peptide Research, Salt Lake City, UT, USA) fluorogenic peptide substrate [4], as described in [13]. The peptide substrate was used at a final concentration of 20 μM. The FLUOstar OPTIMA reader (BMG Labtech, Ortenberg, Germany), equipped with 405 nm excitation and 520 nm emission filters, was used to quantitate fluorescence intensities. Data are presented as means ± SD of three independent experiments.

### 4.5. Western Blot Analysis

After the addition of 5× Sample Buffer (250 mM Tris-HCl, pH 6.8, 5 mM EDTA, 10% SDS, 30% glycerol, and 50 mM DTT), samples were boiled for 5 min and fractionated by SDS–polyacrylamide gel electrophoresis (10% and 12% gels were used for the analyses of phytaspase and 15% gels for SP_Pro samples, respectively). Separated proteins were electrophoretically transferred onto polyvinylidene fluoride (PVDF) membrane (Bio-Rad Laboratories, Hercules, CA, USA). Western blot analyses were performed using rabbit polyclonal anti-tRFP antibodies (Evrogen, Moscow, Russia; at 1:5000 dilution) and secondary anti-rabbit IgG linked to horseradish peroxidase (Thermo Scientific, Waltham, MA, USA). SP_Pro protein was visualized using HisProbe reagent (Thermo Scientific, Waltham, MA, USA). Chemiluminescence detection was performed with ECL Western Lightning Plus reagent (PerkinElmer, Shelton, CT, USA) using the ChemiDoc Imaging System (Bio-Rad Laboratories, Hercules, CA, USA).

## Data Availability

Data are contained within the article and Supplementary Materials.

## Supplementary Materials

The following supporting information can be downloaded at https://www.mdpi.com/article/10.3390/ijms25126729/s1, Figure S1: The S/A *Nt*Phyt mutant lacks the phytaspase-specific proteolytic activity. Table S1: List of the primers used in this study.
