# Supplementary material for: Phytaspase Does Not Require Proteolytic Activity for Its Stress-Induced Internalization
Source: Int J Mol Sci. 2024 Jun 19;25(12):6729. doi: 10.3390/ijms25126729 (PMC11203471; doi:10.3390/ijms25126729)
Supplement: Supplementary file 1 [file ijms-25-06729-s001.zip › ijms-3020072-supplementary.pdf]

Figure S1. The S/A *Nt*Phyt mutant lacks the phytaspase-specific proteolytic activity.

The ΔPro_S/A mutant was produced in *N. benthamiana* leaves either alone (ΔPro_S/A) or in combination with the SP_Pro construct (ΔPro_S/A + SP_Pro). The proteolytic activity was determined in the apoplastic (Ap), intracellular (ICF), and total (T) fractions of leaf extracts using 20 μM Ac-VEID-AFC as a substrate. The data for extracts from leaves producing the 'Native' *Nt*Phyt and from control leaves (Vector) are given for comparison.

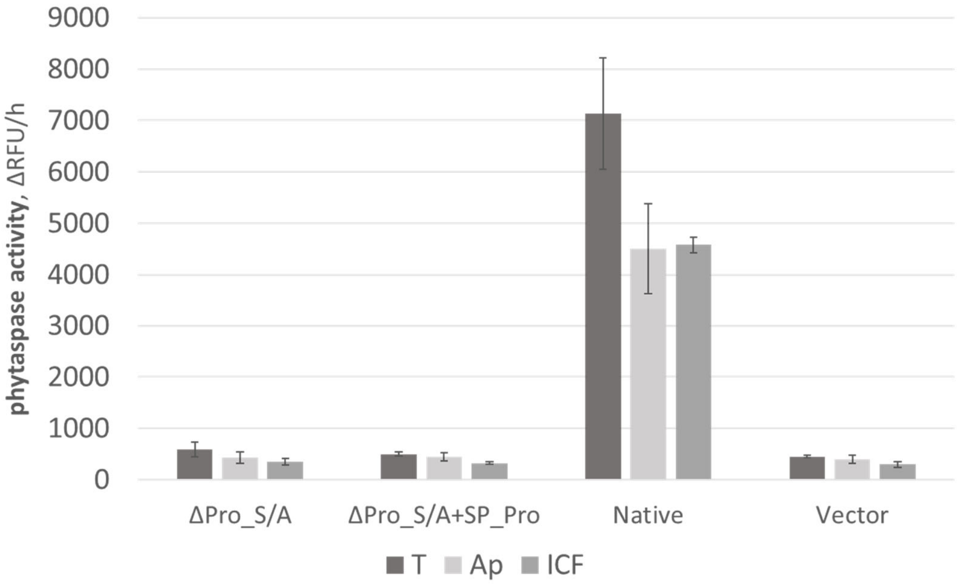

Table S1. List of the primers used in this study.

#	Primer name	Sequence
1	Bam_His_mRFP_dir	GTGGATCCTCTGCACCATCATCATCACCATATGGCCTCCTCCGAGGAC
2	mRFP_Sac(z)_rev	GGTGAGCTCTAAGCTCCGGTGGAGTGGC
3	pLH_seq_dir	GACCTCGAGAATTCTCAAC
4	deltaPro_rev	CGTGTGTGTCGTGGCCATAATAAATGGATTTAG
5	deltaPro_dir	CCATTTATTATGGCCACGACACACACGTCTCAATTC
6	Sub_rev3-1	GTCAGATAACATTGTACCCTCA
7	SP_His_Pro_dir	CCATTTATTATGGCCCAGTCACACCATCATCATCACCATCAGTCAGAAACTTATGTCATCC
8	Prodomain_Sac_rev	CCAGAGCTCAGTCACTTTTAACTGTCATGTCC
9	SP_middle_dir	CCTTATATTTCTTGTTCCTTGCTATCTTACTTACTCTAAATCCATTTATTATGGCCCAGTC
10	SP_Nco_dir_ver2	CACCATGGCCAATTGTATTACCTTATATTTCTTGTTCC
